# Inflammation-based prognostic system predicts postoperative survival of esophageal carcinoma patients with normal preoperative serum carcinoembryonic antigen and squamous cell carcinoma antigen levels

**DOI:** 10.1186/s12957-016-0878-5

**Published:** 2016-05-05

**Authors:** Qilong Ma, Wengao Liu, Ran Jia, Feng Jiang, Hao Duan, Peng Lin, Lanjun Zhang, Hao Long, Hongyun Zhao, Guowei Ma

**Affiliations:** Sun Yat-sen University Cancer Center, State Key Laboratory of Oncology in South China, Collaborative Innovation Center for Cancer Medicine, Guangzhou, China; Guangdong Esophageal Cancer Institute, Guangzhou, China; Department of Thoracic Surgery, Sun Yat-sen University Cancer Center, Guangdong Esophageal Cancer Institute, State Key Laboratory of Oncology in South China, Collaborative Innovation Center for Cancer Medicine, 651 Dongfengdong Road, Guangzhou, China; Department of Medical Oncology, Sun Yat-sen University Cancer Center, State Key Laboratory of Oncology in South China, Collaborative Innovation Center for Cancer Medicine, 651 Dongfengdong Road, Guangzhou, China

**Keywords:** Esophageal carcinoma, Carcinoembryonic antigen, Squamous cell carcinoma antigen, Postoperative survival, Glasgow Prognostic Score

## Abstract

**Background:**

The Glasgow Prognostic Score (GPS) is an established inflammation-based system that is used to predict the prognosis for several types of malignancies. In this retrospective study, we assessed the postoperative survival of 725 patients with non-metastatic esophageal squamous cell carcinoma who had normal preoperative serum tumor marker levels according to the GPS.

**Methods:**

Among 1394 patients who underwent esophagectomy between August 2006 and December 2010, 725 with normal preoperative serum levels of carcinoembryonic antigen (CEA) and squamous cell carcinoma antigen (SCC-Ag) were enrolled. All demographic, pathologic, and survival data were analyzed retrospectively. Uni- and multivariate analyses were performed to evaluate the relationship with overall survival. The Kaplan–Meier analysis and log-rank tests were used to compare the survival curves between patients with GPS 0 (group A) and 1 or 2 (group B).

**Results:**

Patients in group A exhibited significantly better 3- and 5-year cancer-specific survival (CSS) rates (0.780 and 0.759, respectively) than those in group B (0.624 and 0.605, respectively). Multivariate Cox regression analysis revealed that age, tumor length, pathological tumor-node-metastasis (pTNM) stage, venous invasion, lymph node metastasis, serum albumin and C-reactive protein levels, and GPS were associated with postoperative survival of these patients. Further multivariate analysis confirmed that GPS was an independent prognostic factor. The Kaplan–Meier analysis and log-rank tests demonstrated a significant difference in CSS between groups A and B (*P* = 0.001).

**Conclusions:**

GPS may be a valuable prognostic indicator for esophageal cancer patients with normal preoperative CEA and SCC-Ag serum levels.

## Background

Esophageal cancer is one of the most common malignancies worldwide, ranking sixth in terms of cancer-related mortality [1]. It is prevalent in China, Iran, South Africa, Uruguay, France, and Italy. However, almost half of new esophageal cancer cases occur in China, resulting in the highest mortality rate [2]. Importantly, squamous cell carcinoma, which accounts for >95 % of esophageal cancer cases, is the major histological subtype in China [3]. Despite improvements in less invasive treatment strategies, surgery remains the mainstay of curative management. Unfortunately, the outcome of surgical resection for esophageal cancer remains poor with a postoperative 5-year survival rate of only 20–40 % in China [4].

Multiple tumor markers, such as carcinoembryonic antigen (CEA) and squamous cell carcinoma antigen (SCC-Ag), are widely used in clinical practice to estimate the prognosis of patients with esophageal cancer. At our institution, serum levels of CEA and SCC-Ag are routinely measured in patients with esophageal cancer prior to treatment. However, even patients with metastatic disease may not have elevated serum levels of CEA or SCC-Ag before or after surgery [5–7]. Therefore, these tumor makers cannot be applied widely for the prediction of postoperative survival. On the other hand, inflammation-based prognosis using indicators such as the Glasgow Prognostic Score (GPS) has been shown to be a valuable predictor of survival after surgery [8–10]. Because the GPS is thought to reflect the systemic inflammatory response (SIR) on the basis of hypercytokinemia originating from the interaction between the tumor and the host, there may be significant differences between the prognoses made using GPS and tumor markers [11]. Therefore, we hypothesized that GPS is a useful prognostic indicator of postoperative survival in patients with esophageal cancer, especially in those who have normal preoperative serum CEA and SCC-Ag levels. We tested this hypothesis in a retrospective study of 725 patients who had undergone esophagectomy due to esophageal cancer who had normal preoperative serum CEA and SCC-Ag levels.

## Methods

### Patients

Among 1394 patients who underwent esophagectomy at the Department of Thoracic Surgery of Sun Yat-sen University Cancer Center (Guangzhou, China) between August 2006 and December 2010, a total of 725 patients with esophageal cancer were retrospectively enrolled in the present study. Patients eligible for this cohort study had pathologically confirmed esophageal squamous cell carcinoma (ESCC). In all patients, the preoperative serum levels of CEA and SCC-Ag were ≤5.0 ng/ml and ≤1.5 μg/l, respectively. Each patient underwent esophagectomy. Routine laboratory testing of serum levels of C-reactive protein (CRP), albumin (ALB), and tumor markers, including CEA and SCC-Ag, was performed on the day of admission to exclude any effect associated with interference from successive preoperative examinations [12–15]. Patients were excluded if they had previously received cytotoxic chemotherapy or radiotherapy or had a past or current history of another malignancy. Patients were not eligible if tumors were located at the cervical esophagus or esophagogastric junction or had other histological subtypes of esophageal cancer besides ESCC. None of the patients exhibited clinical evidence of infection or other inflammatory conditions, and none received preoperative chemotherapy or irradiation. All patients were staged according to the 7th Edition of the American Joint Committee on Cancer (AJCC) Cancer Staging Manual. The study protocol was approved by the Ethics Committee of Sun Yat-sen University Cancer Center.

### Surgery

The standard surgical approaches consisted of the Sweet (left thoracotomy and diaphragm incision), the McKeown (right thoracotomy, laparotomy, and neck incision), and the Ivor Lewis (laparotomy and right thoracotomy) procedures. In our institute, the majority of patients underwent the Sweet surgical procedure. In this cohort of patients, thoracoabdominal lymphadenectomy was performed.

### Follow-up

Patients were recommended for follow-up examinations at our outpatient department every 3 months for the first 2 years, every 6 months during the subsequent 3 years, and annually thereafter. Follow-up examinations consisted of history taking, physical examination, barium esophagography, chest radiography, abdominal ultrasonography, cervical ultrasonography, and neck-abdomen CT scans. Patients underwent endoscopy and/or positron emission tomography-CT, if necessary.

### GPS evaluation

GPS was estimated as described previously. In brief, patients with both elevated CRP levels (>1.0 mg/dl) and hypoalbuminemia (<3.5 g/dl) were allocated a GPS of 2. Patients with only one of these biochemical abnormalities were allocated a GPS of 1, and those with neither abnormality were allocated a GPS of 0.

For analysis, each patient was allocated to one of two groups: group A (GPS = 0) or group B (GPS = 1 or 2). Patients with a GPS of 1 or 2 were combined because only nine patients had a GPS of 2.

### Statistical analysis

Statistical analysis was performed using SPSS Statistics 18.0 software (IBM SPSS, Inc., Chicago, IL, USA). Odds ratios (OR) with 95 % confidence intervals (95 % CIs) were calculated by multivariate logistic regression analysis. Multivariate analysis was performed to evaluate the influences of sex, age, maximum tumor diameter, smoking history, history of alcohol consumption, intraoperative blood loss, venous invasion, lymph node metastasis, serum CRP, CEA and SCC-Ag levels, pathological tumor-node-metastasis (pTNM) stage, and GPS on cancer-specific survival (CSS). A two-sided *P* < 0.050 was considered statistically significant.

The most valuable prognostic factors identified by univariate analysis were confirmed by multivariate analysis. Multivariate Cox regression analysis was used to exclude other confounding factors affecting survival. The selected variable with univariate analysis was *P* < 0.05. Similarly, Kaplan–Meier analysis and the log-rank tests were used to compare survival curves between groups. Cases were censored at death or the end of follow-up.

## Results

Of the 725 enrolled patients, 539 (74.3 %) were males and 186 (25.7 %) were females, with an age range from 32 to 80 years (median, 58 years). The mean time from the operation to the last censoring date was 28 months. There were no significant differences between groups A and B in terms of most patient characteristics, with the exceptions of the age (*P* = 0.019) and tumor length (*P* < 0.01), as shown in Table 1.

**Table 1:** Baseline characteristics of patients grouped according to the Glasgow Prognostic Score

Characteristic	Group A	Group B	*P*
	(*n* = 616, 85 %)	(*n* = 109, 15 %)	(*χ* ^2^ test)
Age (years)			
≤65	564 (77.79 %)	92 (12.69 %)	
>65	52 (7.17 %)	17 (2.34 %)	0.019
Sex			
Male	452 (62.34 %)	87 (12.00 %)	
Female	164 (22.62 %)	22 (3.03 %)	0.156
Smoking history			
Yes	377 (52.00 %)	75 (10.34 %)	
No	239 (32.97 %)	34 (4.69 %)	0.131
History of alcohol consumption			
Yes	205 (28.28 %)	45 (6.21 %)	0.091
No	411 (56.69 %)	63 (8.69 %)	
Tumor length (cm)			
≤5	474 (65.38 %)	52 (7.17 %)	<0.001
>5	132 (18.21 %)	57 (7.86 %)	
Intraoperative blood loss (ml)			
≤200	471 (64.97 %)	77 (10.62 %)	
>200	143 (19.72 %)	31 (4.28 %)	0.225
pTNM stage			
I	92 (12.69 %)	11 (1.52 %)	
II	299 (41.24 %)	46 (6.34 %)	
III	225 (31.03 %)	52 (7.17 %)	0.069
Venous invasion			
Absence	603 (83.17 %)	107 (14.76 %)	
Presence	13 (1.79 %)	2 (0.28 %)	0.852
Lymph node metastasis			
Absence	332 (45.79 %)	56 (7.72 %)	
Presence	284 (39.17 %)	53 (7.31 %)	0.627

**Fig. 1:**
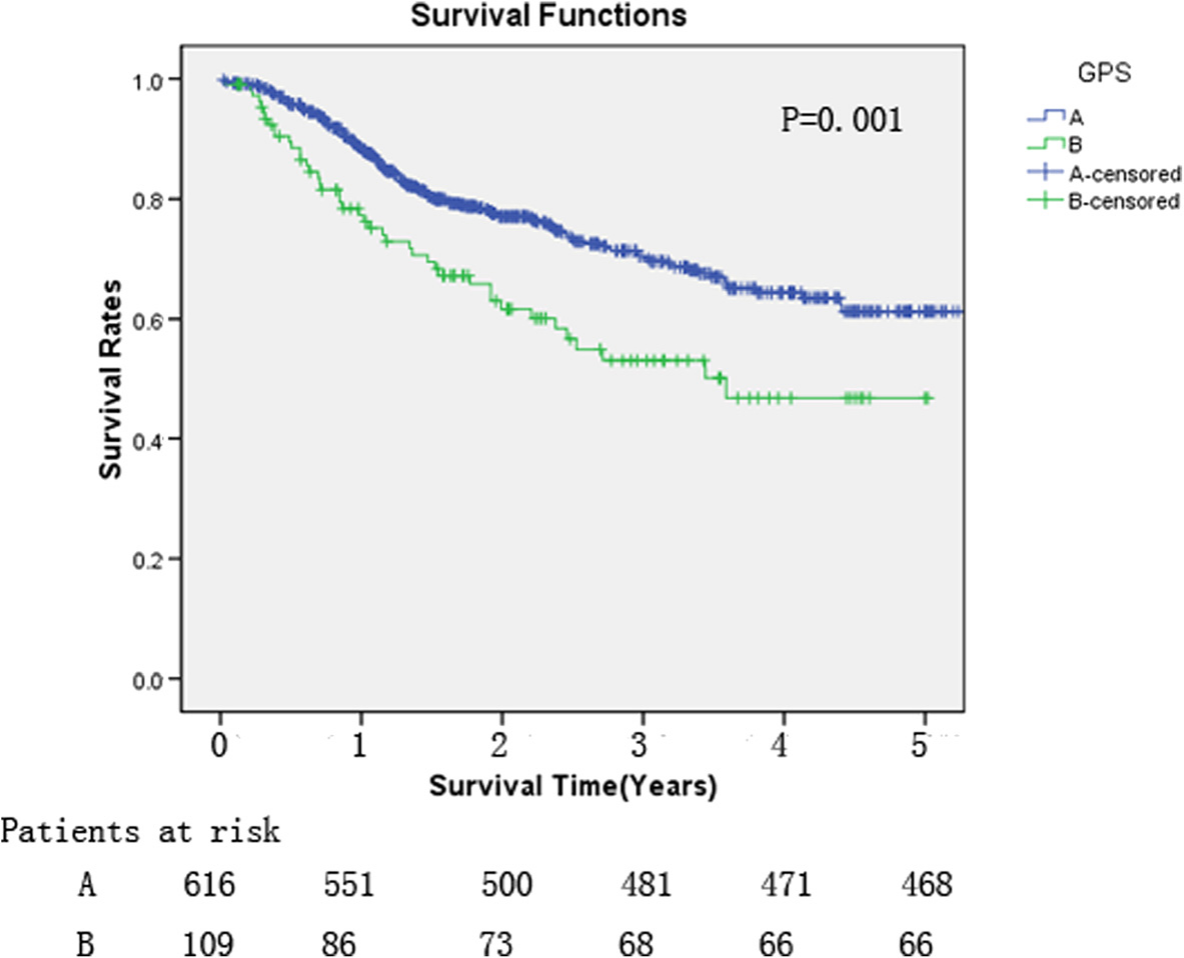
Relationship between patient groups A and B (divided according to the Glasgow Prognostic Score (GPS) and the 5-year cancer-specific survival (CSS). The 5-year CSS of group A (GPS = 0) was significantly higher than that of group B (GPS = 1, 2) (75.9 and 60.5 %, respectively; *P* = 0.001)

To facilitate our analysis, the patients with a GPS of 1 or 2 were combined as a single group (group B) because only nine patients had a GPS of 2. Group A (GPS = 0) comprised 616 patients, and group B (GPS = 1 or 2) comprised 109 patients. Our results indicated that cancer-specific survival (CSS) in group A was significantly higher than that in group B (*P* = 0.001; Fig. 1). Patients in group A exhibited significantly better 3- and 5-year survival rates (0.780 and 0.759, respectively) than those in group B (0.624 and 0.605, respectively).

Univariate and multivariate analyses were performed to identify correlations between clinical characteristics and CSS. As shown in Table 2, univariate analyses indicated that the following clinical characteristics were significant prognostic factors for CSS in patients with esophageal cancer: age [hazard risk (HR) = 1.594; 95 % CI = 1.030–2.467; *P* = 0.036]; tumor length (HR = 1.477; 95 % CI = 1.085–2.011; *P* < 0.013); pTNM stage (HR = 2.511; 95 % CI = 1.966–3.208; *P* < 0.001); venous invasion (HR = 2.984; 95 % CI 1.466–6.072; *P* = 0.003); lymph node metastasis (HR = 2.614; 95 % CI = 1.941–3.519; *P* < 0.001); serum levels of ALB (HR = 3.905; 95 % CI = 1.919–7.945; *P* < 0.001); CRP (HR = 1.751; 95 % CI = 1.239–2.473; *P* = 0.001); and GPS (HR = 0.555; 95 % CI = 0.395–0.779; *P* = 0.001).Further multivariate analysis based on the previously mentioned parameters confirmed that only pTNM stage (HR = 2.419; 95 % CI = 1.892–3.094; *P* < 0.001), venous invasion (HR = 2.462; 95 % CI = 1.207–5.032; *P* = 0.013), and GPS (HR = 1.625; 95 % CI = 1.155–2.286; *P* = 0.005) were independent prognostic factors (Table 3). Our study revealed that GPS, measured at admission prior to treatment, is a useful predictor of postoperative outcome in this group of patients.

**Table 2:** Univariate analysis of factors related to esophageal cancer survival

Characteristic	*P*	HR	95 % CI for Exp(B)	
			Lower	Upper
Age (≤65 or >65 years)	0.036	1.594	1.030	2.467
Sex (male or female)	0.072	0.732	0.521	1.028
Smoking history	0.164	1.238	0.917	1.672
History of alcohol consumption (ml)	0.311	1.164	0.868	1.562
Tumor length (cm)	0.013	1.477	1.085	2.011
Intraoperative blood loss (ml)	0.124	1.279	0.935	1.751
pTNM stage	<0.001	2.511	1.966	3.208
Venous invasion	0.003	2.984	1.466	6.072
Lymph node metastasis	<0.001	2.614	1.941	3.519
Albumin (g/l)	<0.001	3.905	1.919	7.945
CRP (mg/l)	0.001	1.751	1.239	2.473
CEA (ng/ml)	0.070	1.126	0.990	1.281
SCC (μg/l)	0.376	1.176	0.822	1.682
GPS	0.001	0.555	0.395	0.779

**Table 3:** Multivariate analysis of selected clinical characteristics in relation to overall survival

Characteristic	*P*	HR	95 % CI for Exp(B)	
			Lower	Upper
Age (years)	0.068	1.732	1.118	2.684
Tumor length (cm)	0.836	1.339	0.981	1.828
pTNM stage	<0.001	2.419	1.892	3.094
Venous invasion	0.013	2.462	1.207	5.032
Lymph node metastasis	0.339	2.577	1.910	3.477
GPS	0.005	1.625	1.155	2.286

## Discussion

The prognosis for esophageal carcinoma patients is influenced by various pathological characteristics. The tumor markers CEA, SCC-Ag, CA19-9, and CYFRA 21-1 are commonly used to estimate the preoperative tumor status as well as postoperative survival and recurrence in patients with esophageal cancer [5, 6, 16]. Before 2010, the serum levels of CEA and SCC-Ag were measured routinely prior to treatment in all patients at our institution. Therefore, CEA and SCC-Ag were adopted as the two prognostic markers in the present study. However, many patients with metastatic disease may not have elevated CEA or SCC-Ag serum levels before or after surgery [5–7]. This study was undertaken to investigate whether GPS, measured prior to treatment, is useful in predicting the postoperative survival of patients with esophageal cancer who have normal preoperative serum CEA and SCC-Ag levels. Of interest, multivariate analyses revealed that GPS was associated with postoperative survival, indicating that GPS, measured at admission prior to treatment, is a useful predictor of postoperative outcome.

Recently, several reports have indicated that the presence of a systemic inflammatory response is a useful indicator of outcome among patients with esophageal cancer [17–21]. However, few studies have investigated whether GPS is useful for predicting postoperative outcome in ESCC patients with normal preoperative serum CEA and SCC-Ag levels.

The causal relationship between GPS and cancer survival may be due to the presence of a systemic inflammatory response and the influence of the associated nutritional decline [22, 23]. GPS may reflect hypercytokinemia resulting from immune cell activation as part of tumor versus host response, which may differ from the mechanism of tumor progression indicated by tumor marker levels [24, 25]. Due to the presence of cachexia caused by SIR-induced hypercytokinemia, patients in group B were expected to exhibit fewer clinical characteristics on the basis of nutritional status, neutrophil/lymphocyte ratio, and serum ALB level than those in group A.

As mentioned previously, GPS appears to be a significant prognostic factor that can be used to facilitate an accurate estimation of tumor characteristics in select populations. The results of our study showed that CSS of group A was superior to that of group B. Interestingly, univariate analysis identified two types of clinical characteristics as significant prognostic factors for CSS in patients with esophageal cancer: tumor-related characteristics, such as lymph node metastasis, tumor length, venous invasion, lymph node metastasis and pTNM stage (I, II, and III), and SIR-related characteristics, such as the serum levels of ALB and CRP and the GPS (0/1, 2) [26]. It is well-known that the latter three indicators are associated with hypercytokinemia. Moreover, among the clinical characteristics identified in the univariate analysis, multivariate analysis clearly demonstrated that the inflammation-based prognostic indicator GPS was associated with CSS. Although there were three SIR-related characteristics which were associated with CSS, Kaplan–Meier analysis revealed that patients could be divided into two independent groups on the basis of GPS (GPS 0/1, 2).

## Conclusions

The levels of CRP, albumin, CEA, SCC-Ag, and other serum indexes are routinely measured in patients. Consequently, it is simple to evaluate the GPS for each patient prior to treatment. Thus, the pretreatment GPS is an easily measurable and valuable preoperative prognostic indicator for esophageal cancer patients with normal preoperative CEA and SCC-Ag serum levels.
